# TagR, a newly identified member of the MarR family of transcriptional regulators, represses the NRPS operon in *Klebsiella oxytoca*

**DOI:** 10.1128/spectrum.01853-26

**Published:** 2026-08-05

**Authors:** Cristopher Perez, Mario A. López-Luis, Carlos J. Jiménez-Sánchez, Fermín I. Hernández-Pompa, Diana Rodríguez-Valverde, Sandra Rivera-Gutiérrez, Roberto Rosales-Reyes, Fernando Chimal-Cázares, Jorge Soria-Bustos, Jorge A. Yañez-Santos, Maria L. Cedillo, Miguel A. De la Cruz, James G. Fox, Miguel A. Ares

**Affiliations:** 1 Posgrado en Ciencias en Biomedicina y Biotecnología Molecular, Escuela Nacional de Ciencias Biológicas, Instituto Politécnico Nacional 61735, Mexico City, Mexico; 2 Unidad de Investigación Médica en Enfermedades infecciosas y Parasitarias, Hospital de Pediatría, Centro Médico Nacional Siglo XXI, Instituto Mexicano del Seguro Social, Mexico City, Mexico; 3 Departamento de Microbiología, Escuela Nacional de Ciencias Biológicas, Instituto Politécnico Nacional, Mexico City, Mexico; 4 Laboratorio de Biotecnología y Boinformática Genómica, Departamento de Bioquímica, Escuela Nacional de Ciencias Biológicas, Instituto Politécnico Nacional, Mexico City, Mexico; 5 Unidad de Medicina Experimental, Facultad de Medicina, Universidad Nacional Autónoma de México, Mexico City, Mexico; 6 División de Ciencias de la Salud, Universidad Tecnológica de Tulancingo, Tulancingo, Hidalgo, Mexico; 7 Centro de Detección Biomolecular, Benemérita Universidad Autónoma de Puebla, Puebla, Mexico; 8 Facultad de Medicina, Benemérita Universidad Autónoma de Puebla, Puebla, Mexico; 9 Division of Comparative Medicine, Massachusetts Institute of Technology, Cambridge, Massachusetts, USA; Illinois State University, Normal, Illinois, USA

**Keywords:** TagR, MarR family regulator, NRPS operon, *Klebsiella oxytoca*

## Abstract

Toxigenic *Klebsiella oxytoca* strains produce the pyrrolobenzodiazepine enterotoxins tilimycin (TM) and tilivalline (TV), which contribute to the development of antibiotic-associated hemorrhagic colitis. The biosynthesis of these toxins depends on the nonribosomal peptide synthetase (NRPS) operon located within the *til* pathogenicity island. Although several global and signal-responsive regulators of NRPS operon expression have been identified, the regulatory network governing enterotoxin biosynthesis remains incompletely characterized. In this study, we identified a previously unrecognized transcriptional regulator encoded within the *til* pathogenicity island of *K. oxytoca*. This protein, designated TagR (Tilivalline-associated genes repressor), is a member of the MarR family and acts as a negative regulator of NRPS operon expression. Structural prediction, molecular dynamics simulations, and biochemical analyses demonstrated that TagR exhibits the characteristic architecture of MarR family regulators and forms a stable homodimer. Deletion of *tagR* led to significant upregulation of the NRPS-associated genes *npsA*, *thdA*, and *npsB*, while complementation restored transcriptional repression. Electrophoretic mobility shift assays confirmed that TagR binds directly and specifically to the regulatory region upstream of the NRPS operon, supporting a mechanism of direct transcriptional repression. Consistent with these findings, loss of TagR significantly increased the cytotoxicity of *K. oxytoca* culture supernatants toward HeLa cells. Collectively, these results identify TagR as a direct repressor of the NRPS operon and expand the regulatory framework governing enterotoxin biosynthesis in toxigenic *K. oxytoca*. This study provides new insight into the transcriptional control of virulence-associated genes and establishes TagR as a previously unrecognized component of the regulatory network controlling TM and TV production.

**IMPORTANCE:**

Elucidating the mechanisms by which toxigenic *Klebsiella oxytoca* regulates enterotoxin production is critical for understanding the pathogenesis of antibiotic-associated hemorrhagic colitis. TagR is identified as a previously unrecognized MarR family regulator that directly represses the nonribosomal peptide synthetase (NRPS) operon responsible for tilimycin (TM) and tilivalline (TV) biosynthesis. This discovery uncovers a novel regulatory mechanism governing toxin production and offers new perspectives on virulence regulation in this emerging intestinal pathogen.

## INTRODUCTION

Toxigenic strains of *Klebsiella oxytoca* are significant causative agents of antibiotic-associated hemorrhagic colitis (AAHC), an intestinal disease characterized by abdominal pain, bloody diarrhea, and mucosal injury following antimicrobial therapy (1). Antibiotic administration disrupts gut microbiota composition and stability, resulting in intestinal dysbiosis and reduced colonization resistance. These conditions facilitate the proliferation of toxigenic *K. oxytoca* strains and their pathogenic interactions with the intestinal epithelium (2). Recent studies have focused on identifying the molecular determinants of *K. oxytoca* virulence, particularly those involved in enterotoxin biosynthesis and regulation (3–8).

The pathogenicity of toxigenic *K. oxytoca* strains is primarily due to the production of the pyrrolobenzodiazepine enterotoxins tilimycin (TM) and tilivalline (TV), two structurally related secondary metabolites with potent cytotoxic effects on intestinal epithelial cells (9). TM functions as a genotoxic agent that induces DNA damage and cell cycle arrest, whereas TV stabilizes microtubules and disrupts mitotic progression (2). Together, these toxins compromise epithelial barrier integrity, disrupt tight junctions, and contribute to the intestinal inflammation and tissue damage characteristic of AAHC (10).

TM and TV biosynthesis depend on a nonribosomal peptide synthetase (NRPS) operon located within the *til* pathogenicity island of toxigenic *K. oxytoca* (9, 11). This operon, comprising the *npsA*, *thdA*, and *npsB* genes, is essential for enterotoxin production and cytotoxic activity (9). Although the biosynthetic pathway responsible for TM and TV production has been extensively characterized, recent evidence indicates that expression of the NRPS operon is regulated by a complex network. Studies have demonstrated that the global regulators CRP, Lrp, Fis, and IHF, as well as the quorum-sensing regulator SdiA, positively influence genes involved in TM/TV biosynthesis, thereby increasing cytotoxicity toward epithelial cells. In contrast, the response regulator OmpR acts as a negative regulator of these virulence-associated genes. These findings collectively indicate that the NRPS operon expression is precisely coordinated by multiple regulatory pathways that integrate nutritional, environmental, physiological, and interspecies signaling cues (3–7).

While several global regulators of enterotoxin production have been identified, local transcriptional regulators directly associated with the NRPS locus have not yet been characterized. Understanding the mechanisms by which toxigenic *K. oxytoca* regulates enterotoxin production is critical for elucidating host colonization, epithelial injury, and disease progression.

Bacterial adaptation to changing environmental and host-associated conditions depends on complex transcriptional regulatory networks that coordinate gene expression in response to physiological and environmental cues (12–14). These networks enable bacteria to optimize metabolism, respond to stress, and modulate virulence-associated processes during infection and adaptation (15–18). Among the most prevalent bacterial transcriptional regulators are members of the MarR family (Multiple Antibiotic Resistance Regulator), a group of DNA-binding proteins conserved in both gram-positive and gram-negative bacteria (19–21).

MarR family proteins participate in diverse cellular functions, including antibiotic resistance, oxidative stress adaptation, metabolism, environmental sensing, and regulation of virulence genes (21–23). Structurally, these proteins typically act as homodimeric transcriptional regulators with winged helix-turn-helix (wHTH) DNA-binding motifs that recognize promoter regions of target genes and frequently mediate transcriptional repression (21, 23, 24).

In multiple bacterial pathogens, MarR family regulators control virulence determinants, pathogenicity island-associated genes, and specialized metabolic pathways that enhance bacterial fitness and host interactions (20, 25–27). In addition to their established roles in stress adaptation and antimicrobial resistance, these regulators are increasingly recognized as central components of transcriptional networks underlying bacterial pathogenicity (21, 28–30). However, the role of MarR family proteins in regulating enterotoxin biosynthesis in toxigenic *K. oxytoca* remains uninvestigated.

This study examines a MarR family transcriptional regulator encoded within the *til* pathogenicity island of toxigenic *K. oxytoca*. The findings indicate that this regulator, designated TagR (Tilivalline-associated genes repressor), functions as a negative regulator of the NRPS operon responsible for TM and TV biosynthesis. These results offer new insights into the transcriptional regulation of enterotoxin-associated genes and identify TagR as an additional component of the complex regulatory network controlling NRPS operon expression and enterotoxin production in toxigenic *K. oxytoca*. More broadly, this study advances understanding of the virulence-associated regulatory mechanisms that govern pathogenicity island-encoded functions in this significant enteric pathogen.

## MATERIALS AND METHODS

### Protein structure modeling and equilibrium molecular dynamics simulations

The amino acid sequence employed for structural modeling of the TagR protein was retrieved from the NCBI database (HMPREF9685_00563) and corresponds to the wild-type strain of *Klebsiella oxytoca* MIT 09-7231 (GCF_001078175.1). The putative TagR homodimer structural model was generated using the AlphaFold 3 server (31) with default settings. Model selection was based on the predicted local distance difference test (pLDDT) score. The selected TagR homodimer model was prepared for equilibrium molecular dynamics (EMD) simulations using the CHARMM-GUI web server (32), employing default parameters, explicit solvent, and a simulation box extending 10 Å from the protein surface.

Equilibrium molecular dynamics simulations were performed using NAMD3 software v3.01 (33) for 40 ns. Trajectories were analyzed with the MDAnalysis Python3 library (34) to calculate root mean square deviation (RMSD) and root mean square fluctuation (RMSF), facilitating assessment of structural stability and local flexibility of the TagR homodimer. Inter-chain interaction energy was calculated using NAMD2 and VMD v1.9.3 (35). Data visualization was conducted with the ggplot2 package (36).

### Identification and multiple sequence alignment of TagR homologs

The conserved wHTH domain of TagR was identified by conducting BLASTP searches against the RefSeq database (37) to find homologous MarR family proteins. MarR family proteins exhibiting the highest sequence identity to TagR were selected for multiple sequence alignment using the BLAST server. The resulting alignment was subsequently edited and visualized with Jalview v2.11.5.1 (38).

### Assessment of His_6_-TagR purity and experimental validation of dimer stability

The purity of recombinant His_6_-TagR and the dimeric state predicted by structural modeling were assessed by analyzing purified protein preparations using SDS-PAGE and 05SAR-PAGE. SDS-PAGE verified protein purity under denaturing conditions, while 05SAR-PAGE evaluated the stability of the predicted TagR dimer under mild detergent conditions that preserve non-covalent protein-protein interactions.

For SDS-PAGE, purified His_6_-TagR was mixed with 4× loading buffer containing 277.8 mM Tris-HCl (pH 6.8), 44.4% glycerol, 4.4% SDS, 50 mM β-mercaptoethanol, and 0.02% bromophenol blue, then incubated at 95°C for 10 min. Proteins were separated using a discontinuous polyacrylamide gel system with a 15% resolving gel in 375 mM Tris-HCl (pH 8.8) and a 6% stacking gel in 125 mM Tris-HCl (pH 6.8) (39, 40). Both gels contained 0.1% SDS and were polymerized with ammonium persulfate (APS) and N,N,N′,N′-tetramethylethylenediamine (TEMED). Electrophoresis was conducted at a constant voltage of 100 V for 120 min using Tris-glycine-SDS running buffer (25 mM Tris, 250 mM glycine, and 0.1% SDS). Protein molecular masses were determined using a PageRuler Prestained Protein Ladder (Thermo Fisher Scientific).

05SAR-PAGE was performed as previously described (41) with minor modifications. The discontinuous gel system described above was used, except that 0.05% sodium lauroyl sarcosinate (sarkosyl) replaced SDS in both the stacking and resolving gels. Protein samples were mixed 1:1 with loading buffer containing 250 mM Tris-HCl (pH 6.8), 50% glycerol, 1% bromophenol blue, and 0.05% sarkosyl.

For dimer stability analyses, purified His_6_-TagR was analyzed either untreated or after treatment with reducing or denaturing agents prior to electrophoresis. For the treatments, aliquots of the purified protein were mixed with loading buffer alone (untreated) or supplemented with 5% β-mercaptoethanol, 50 mM dithiothreitol (DTT), or 1% SDS. SDS-treated samples were then heated at 95°C for 10 min prior to electrophoresis, whereas untreated, β-mercaptoethanol-treated, and DTT-treated samples were loaded without heating. All samples were subsequently analyzed by 05SAR-PAGE under the conditions described above.

Electrophoresis was conducted in running buffer containing 25 mM Tris, 250 mM glycine, and 0.05% sarkosyl. Samples were initially electrophoresed at 80 V for approximately 30 min to facilitate entry into the resolving gel, after which the voltage was increased to 120 V. Electrophoresis continued at room temperature until the bromophenol blue tracking dye reached the bottom of the gel. Protein molecular masses were determined using a PageRuler Unstained Protein Ladder (Thermo Fisher Scientific).

### Bacterial strains and growth conditions

The toxigenic *K. oxytoca* strain MIT 09-7231 (42), its derived Δ*tagR* mutant, and the complemented strain Δ*tagR* pT3-TagR were utilized throughout this study. Specifically, Table 1 provides a comprehensive list of all bacterial strains and plasmids. To ensure consistency, cultures were routinely grown in tryptone lactose broth (TLB), prepared as previously described (43), containing 17 g/L tryptone, 10 g/L lactose, and 2.5 g/L dipotassium hydrogen phosphate. Furthermore, bacterial cultures were incubated at 37°C with shaking. When necessary, the media were supplemented with antibiotics at the following concentrations: ampicillin (100 μg/mL), kanamycin (50 μg/mL), or tetracycline (10 μg/mL).

**TABLE 1:** Bacterial strains and plasmids used in this study

Strains	Description^*a*^	References
*K. oxytoca* MIT 09-7231	Wild-type *Klebsiella oxytoca* strain MIT 09-7231, Amp^R^	(42)
*K. oxytoca ∆tagR*	*K. oxytoca* ∆*tagR::Kan*, Kan^R^	This study
*K. oxytoca* ∆*tagR* pT3-TagR	*K. oxytoca* ∆*tagR::Kan* pT3-TagR, Kan^R^, Tet^R^ (*trans*-complemented)	This study
*K. oxytoca ∆npsA*	*K. oxytoca* Δ*npsA*::FRT	(3)
*E. coli* MC4100	Cloning strain *F^−^ araD139*∆(*argF-lac*) *U169 rspL150 relA1 flbB5301 fruA25 deoC1 ptsF25*	(44)
*E. coli* BL21 (DE3)	F^−^ *omp*T *hsd*S_B_(r_B_^−^, m_B_^−^) *gal dcm* (DE3)	Invitrogen

Plasmids	Description	References
pKD119	pINT-ts derivative containing the λ-Red recombinase system under an arabinose-inducible promoter, Tet^R^	(45)
pKD4	pANTsy derivative template plasmid containing the kanamycin cassette for λ-Red recombination, Amp^R^ Plasmid that shows temperature-sensitive	(45)
pMPM-T3	p15A derivative low-copy-number expression vector, *lac* promoter, Tet^R^	(46)
pT3-TagR	pMPM-T3 derivative expressing *tagR* from the *lac* promoter, Tet^R^	This study
pMPM-T6	p15A derivative expression vector, pBAD (*ara*) promoter, Tet^R^	(46)
pT6-TagR	pMPM-T6 derivative expressing N-terminal His_6_-TagR from the pBAD(*ara*) promoter, Tet^R^	This study

^*a*^ Amp^R^, ampicillin resistance; Kan^R^, kanamycin resistance; Tet^R^, tetracycline resistance.

### Generation of the *tagR* isogenic mutant strain

The toxigenic *K. oxytoca* strain MIT 09-7231 was used as the parental strain for mutagenesis experiment. Bacterial strains and plasmids used in this study are listed in Table 1. An in-frame deletion of *tagR* was generated using the λ Red recombination system as previously described (45).

Briefly, a kanamycin resistance cassette was amplified from plasmid pKD4 using primers containing 45-bp homology extensions corresponding to the regions immediately upstream and downstream of the *tagR* coding sequence (Table 2). The resulting PCR product was purified using the QIAquick PCR Purification Kit (Qiagen) and introduced by electroporation into competent *K. oxytoca* MIT 09-7231 cells harboring plasmid pKD119, which expresses the λ Red recombinase system. Expression of the recombination machinery was induced by supplementing the culture medium with l-(+)-arabinose to a final concentration of 1% prior to preparation of electrocompetent cells.

**TABLE 2:** Primers used in this study^*a*^^,*b*^

Primer	Sequence (5′−3′)	Target gene
For gene deletion		
tagR-H1P1	TATACGGCATCAGGAGAATGTATGGAAATGAATAAAAAAGTGGCTTGTAGGCTGGAGCTGCTTCG	*tagR*
tagR-H2P2	GCTTCATCACCATAAACTTTATTAATTTTCACTGGTATCTTCGAGCATATGAATATCCTCCTTAG	
For mutant characterization		
tagR-MC-F	TTCATTCCTTGAAGGGTTATGGT	*tagR*
tagR-MC-R	AGAGAATACCACGCCAGCG	
For qPCR		
npsA-F	AAATACGTGGCTTCCGCATC	*npsA*
npsA-R	TCCTGCGTGACATAACAAGC	
thdA-F	TGGACAACGTTGAGCAACAG	*thdA*
thdA-R	TGCTTACCATTGACGCCAAC	
npsB-F	TGAGCATTTGCAGCTGGTTC	*npsB*
npsB-R	ATGCGTGGCAACTTTGTGTG	
rrsH-F	CAGGGGTTTGGTCAGACACA	*rrsH*
rrsH-R	GTTAGCCGGTGCTTCTTCTG	
For clone *tagR* into the vector pMPM-T3		
tagR-XhoI-T3-F	GGG**CTCGAG**CTTTTCATTCCTTGAAGGGTTATGGT	*tagR*
tagR-BamHI-T3-R	CCC**GGATCC**ACATTTTAGCAAAATTCCGATGCC	
For clone *tagR* into the vector pMPM-T6		
tagR-NcoI-T6-F	GGG**CCATGG**AA*CATCATCATCATCATCAT*ATGAATAAAAAAGTGGCTAGTGG	*tagR*
tagR-HindIII-T6-R	CCC**AAGCTT**CTTCATCACCATAAACTTTATTAATTTTCAC	
For EMSA		
npsA-EM-1-F	TTATCGCTCCAGAACGGCTG	*npsA*
npsA-EM-100-F	TTTAACAAAAAAGCGATGAGAAAGTGC	
npsA-EM-157-F	ACGATACGGTTTGCACAAAT	
npsA-EM-206-F	TCAAAAAAATTTATATTTGATTTATGT	
npsA-EM-313-F	AATTGACTGGGGATGAGGTTCG	
npsA-EM-234-R	AAACATAAATCAAATATAAATTTTT	
npsA-EM-341-R	CTATCTTCGAACCTCATCCCCA	
npsA-EM-438-R	AATGCCGCCAGCTTACCG	
npsA-EM-513-R	CACTCTCTCCTGGAGAATTAGGA	
pehX-EM-F	AATATGCCTTTACGGTGCGC	*pehX*
pehX-EM-R	GATCGTCTGAACCCAGCAGG	

^*a*^ Underlined sequences indicate regions homologous to the pKD4 template. Restriction enzyme recognition sites are shown in bold. The nucleotide sequence encoding the N-terminal hexahistidine (His_6_) tag is indicated in italics.

^*b*^ Sequences are shown in 5′ to 3′ direction.

Electroporation was performed using a MicroPulser Electroporator (Bio-Rad). Recombinant clones were selected on kanamycin-containing medium and screened by PCR using primers flanking the *tagR* locus. Correct allelic replacement of *tagR* by the kanamycin resistance cassette was subsequently confirmed by sequencing of the PCR amplicons.

### Construction of complementation and expression plasmids

The complementation plasmid pT3-TagR was generated by PCR amplification of the *tagR* coding sequence using primers listed in Table 2. The purified amplicon was digested with XhoI and BamHI, then ligated into the corresponding sites of the low-copy-number expression vector pMPM-T3. In this construct, *tagR* expression was controlled by the vector-encoded *lac* promoter (Plac). The integrity of the recombinant plasmid was verified by PCR and DNA sequencing before introduction into the Δ*tagR* mutant strain for complementation studies.

For recombinant protein production, the *tagR* coding sequence was amplified using a forward primer containing an NcoI restriction site and six histidine codons in-frame with the *tagR* gene, resulting in an N-terminal His₆-tag fusion. The reverse primer included a HindIII restriction site downstream of the coding sequence (Table 2). The PCR product was digested with NcoI and HindIII and cloned into the corresponding sites of the pMPM-T6 expression vector, generating plasmid pT6-TagR. The integrity of the His₆-tag fusion, reading frame, and *tagR* coding sequence was confirmed by PCR and DNA sequencing before recombinant protein expression.

### RNA isolation and reverse transcription-quantitative PCR (RT-qPCR)

Total RNA was isolated using the hot phenol protocol as previously described (47). Residual genomic DNA was eliminated with the TURBO DNA-free kit (Invitrogen). RNA concentration and purity were determined using a NanoDrop ONE spectrophotometer (Thermo Scientific), and RNA integrity was assessed by electrophoresis on 1.5% bleach-denaturing agarose gels (48).

Complementary DNA (cDNA) was synthesized from 1 μg of total RNA using the RevertAid First Strand cDNA Synthesis Kit (Thermo Fisher Scientific) according to the manufacturer’s instructions. Negative control reactions lacking reverse transcriptase were included to assess potential genomic DNA contamination.

Transcript abundance was quantified by real-time quantitative PCR (RT-qPCR) using a LightCycler 480 Instrument (Roche) and SYBR Green I Master Mix (Roche). Each 10-μL reaction contained 5 μL of 2× SYBR Green I Master Mix, 2.5 μL of cDNA template (approximately 25 ng), 0.5 μL of each primer (20 μM), and 1.5 μL of nuclease-free water. Amplification was carried out using FastStart DNA Taq Polymerase provided in the master mix. Product formation was monitored by measuring SYBR Green I fluorescence during each amplification cycle.

The amplification protocol included an initial denaturation at 95°C for 10 min, followed by 45 cycles of 95°C for 10 s, 59°C for 10 s, and 72°C for 10 s. Amplification specificity was assessed by melt-curve analysis, which involved 95°C for 10 s, a continuous temperature increase from 65°C to 97°C, and a final cooling step at 40°C for 10 s. No-template and no-reverse-transcriptase controls were included in all assays.

All reactions were performed in technical triplicate using RNA isolated from three independent biological replicates. Expression levels were normalized to the *rrsH* transcript, which encodes 16S rRNA, and relative transcript abundance was calculated using the 2^−ΔΔC*t*^ method (49, 50). Results are presented as mean values from three independent biological experiments, each performed in triplicate.

### Purification of recombinant TagR protein

To prevent aggregation of recombinant TagR into insoluble inclusion bodies and to maintain the protein in a fully soluble state during purification, His_6_-TagR was purified under strongly denaturing conditions. All extraction and purification buffers were supplemented with 8 M urea, a chaotropic agent that disrupts non-covalent protein-protein interactions and prevents protein aggregation during purification.

The pT6-TagR expression plasmid was introduced into competent *Escherichia coli* BL21(DE3) cells by electroporation. Recombinant His_6_-TagR was purified as previously described (4, 6, 7, 51, 52) with minor modifications. Bacterial cultures harboring pT6-TagR were grown to mid-exponential phase, and protein expression was induced with l-(+)-arabinose for 6 h at 37°C. Cells were harvested by centrifugation and resuspended in denaturing lysis buffer containing 8 M urea, 100 mM Na_2_HPO_4_, and 10 mM Tris-HCl (pH 8.0). The continuous presence of urea throughout purification ensured complete solubilization of His_6_-TagR and facilitated recovery of the recombinant protein by Ni-NTA affinity chromatography.

Following sonication, cell debris was removed by centrifugation, and the clarified lysate was applied to a Ni-NTA agarose affinity column (Qiagen). The column was washed with buffer containing 50 mM imidazole, 10 mM Na_2_HPO_4_, 1.8 mM KH_2_PO_4_, 137 mM NaCl, and 2.7 mM KCl (pH 7.4). The recombinant protein was eluted using the same buffer supplemented with 500 mM imidazole.

Protein fractions were analyzed by SDS-PAGE and visualized using Coomassie Brilliant Blue staining. After Ni-NTA affinity purification, His_6_-TagR was refolded by stepwise dialysis at 4°C using a 12 kDa molecular-weight-cutoff cellulose membrane (Sigma-Aldrich). Urea was removed by sequentially reducing its concentration from 6 to 0 M in a refolding buffer containing 20 mM Tris-HCl (pH 8.0), 50 mM KCl, 1 mM DTT, and 10% glycerol, as previously described for the recovery of recombinant proteins purified under denaturing conditions (7, 53, 54).

Following refolding, samples were centrifuged at 12,000 × *g* for 10 min at 4°C to remove insoluble material, and the soluble protein fraction was collected. Protein concentration was determined using the Bradford assay (Bio-Rad). Purified His_6_-TagR protein was stored at −70°C in storage buffer containing 50% glycerol, 10 mM Na_2_HPO_4_, 1.8 mM KH_2_PO_4_, 137 mM NaCl, and 2.7 mM KCl (pH 7.4).

### Electrophoretic mobility shift assays (EMSAs)

The DNA-binding activity of TagR was assessed using electrophoretic mobility shift assay (EMSA) as previously described (3). Binding reactions were prepared in a final volume of 20 μL with H/S 1× gel-shift buffer containing 40 mM HEPES, 8 mM MgCl_2_, 50 mM KCl, 1 mM DTT, 0.05% NP-40, and 0.1 mg/mL BSA (4). The target probe comprised 100 fmol of a 513-bp DNA fragment representing the intergenic regulatory region upstream of *npsA*, the first gene of the NRPS operon, and was incubated with increasing concentrations of purified His_6_-TagR protein (0–0.6 μM). As a negative control, 100 fmol of a 309-bp DNA fragment from the coding region of the *pehX* gene of *K. oxytoca* was used under identical conditions.

Furthermore, a series of overlapping 5′ and 3′ deletion fragments spanning the *npsA* upstream regulatory region was generated by PCR with specific primer pairs (Table 2) to identify the DNA region required for TagR binding. The resulting PCR products were purified and employed as DNA probes in EMSAs using the same binding and electrophoretic conditions described previously. Reaction mixtures were incubated for 20 min at room temperature and resolved on 6% native polyacrylamide gels in 0.5× Tris-borate-EDTA buffer. DNA bands were detected by ethidium bromide staining and UV transillumination.

### Identification of the *npsA* promoter region

The upstream regulatory sequence of *npsA* was analyzed to identify potential promoter elements. A 467-base pair DNA fragment located immediately upstream of the *npsA* start codon in the *K. oxytoca* MIT 09-7231 genome (GenBank accession number: GCA_001078175.1) was evaluated using the Neural Network Promoter Prediction web server (https://www.fruitfly.org/seq_tools/promoter.html). The resulting promoter architecture prediction was then compared with the experimentally mapped TagR-binding region.

### Lactate dehydrogenase (LDH) cytotoxicity assays

Cytotoxic activity was assessed by quantifying lactate dehydrogenase (LDH) release with the CyQUANT LDH Cytotoxicity Assay Kit (Invitrogen) according to the manufacturer’s protocol. HeLa cells were cultured in Dulbecco’s modified Eagle medium (DMEM) supplemented with 4.5 g/L glucose and 10% fetal bovine serum (FBS). For cytotoxicity assays, cells were seeded in 96-well flat-bottom plates at a density of 1 × 10^4^ cells per well and permitted to adhere before treatment.

Cell-free culture supernatants from wild-type, Δ*tagR*, Δ*tagR* pT3-TagR, and Δ*npsA* strains were prepared by centrifugation followed by filtration through 0.22 μm membranes. To assess cytotoxic activity, 10 μL of each bacteria-free supernatant was combined with 90 μL of HeLa cell culture medium (DMEM). Sterile TLB medium processed identically to bacterial cultures served as a negative control. The Δ*npsA* mutant, which does not produce tilimycin (TM) or tilivalline (TV), was included as an additional control to confirm that cytotoxicity was dependent on NRPS-mediated enterotoxin production. Cells were incubated for 48 h at 37°C in a humidified atmosphere with 5% CO_2_.

After incubation, 50 μL of culture supernatant from each well was transferred to a new 96-well plate and combined with LDH detection reagents as specified by the manufacturer. Absorbance was measured at 490 and 680 nm using a Multiskan Ascent microplate reader (Thermo Fisher Scientific). The 680 nm background absorbance was subtracted from the corresponding 490 nm readings. Spontaneous LDH release was measured with untreated cells, and maximum LDH release was determined with the lysis solution provided in the kit. Cytotoxicity was calculated as a percentage using the following formula:

% Cytotoxicity = [(Experimental LDH release − Spontaneous LDH release)/(Maximum LDH release − Spontaneous LDH release)] × 100

Data are presented as mean values from three independent biological experiments.

### Statistical analysis

Statistical analyses were conducted using GraphPad Prism version 11.0.2 (GraphPad Software, San Diego, CA, USA). Group differences were assessed with one-way ANOVA, followed by Tukey’s comparison test. The data represent three independent experiments performed in triplicate. Differences with *P* values less than 0.05 were considered statistically significant.

## RESULTS

### Structural and stability prediction of the putative TagR homodimer

**Fig 1:**
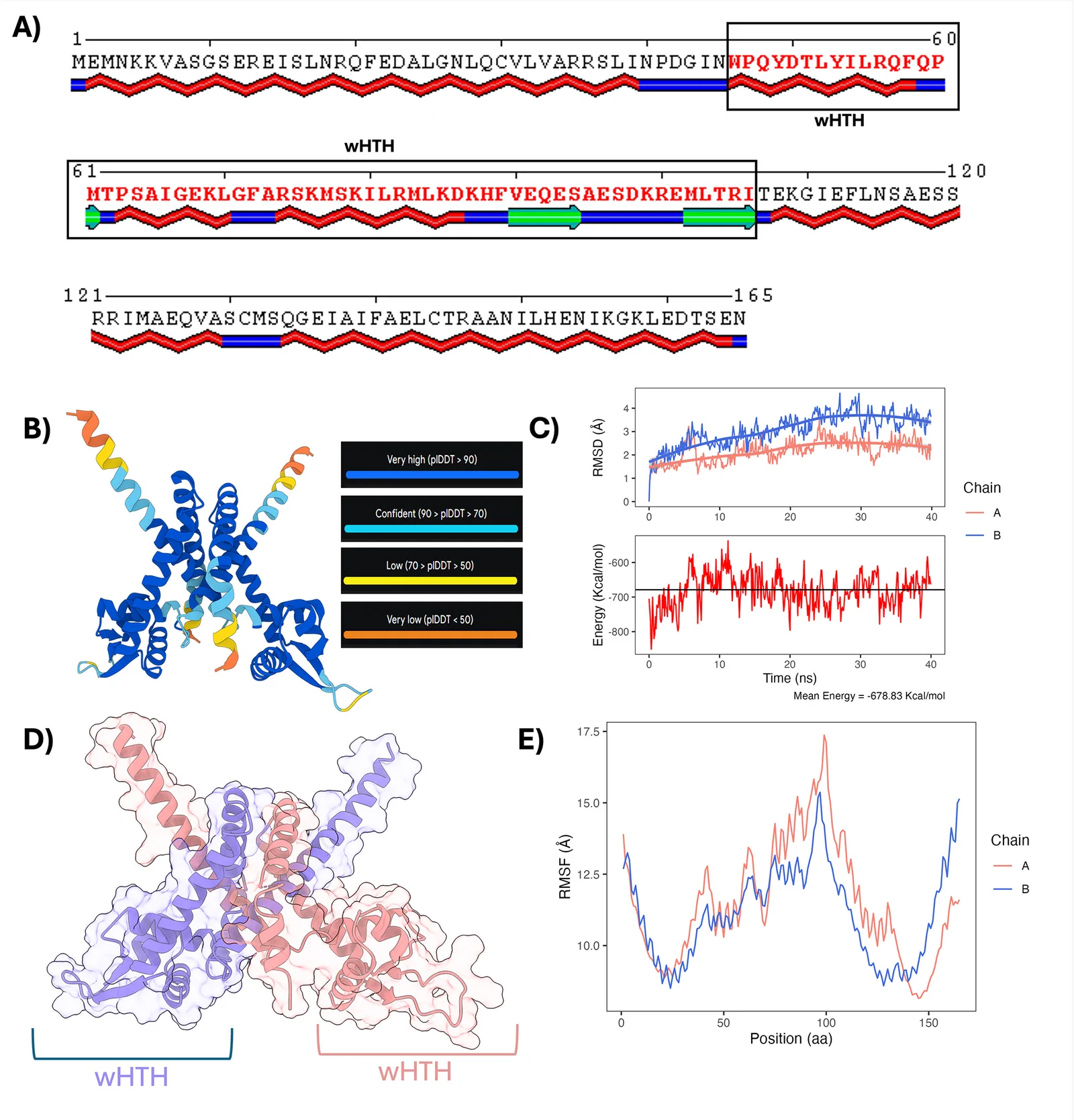
Structural prediction and stability assessment of the *Klebsiella oxytoca* TagR homodimer. (**A**) Amino acid sequence of TagR showing the predicted secondary structure, including α-helices (red coils), β-strands (green arrows), and coil regions (blue lines). The winged helix-turn-helix (wHTH) DNA-binding domain is indicated. (**B**) Initial structural model predicted by AlphaFold, with residues colored according to the per-residue confidence score, pLDDT (predicted local distance difference test). (**C**) Analysis of the 40 ns EMD simulation. The upper panel shows the RMSD (root-mean-square deviation) trajectory, indicating structural convergence over the final 20 ns. The lower panel shows the inter-chain interaction energy over the course of the simulation. (**D**) Refined TagR homodimer structure after EMD, depicted in ribbon and surface representations; chains A and B are colored red and blue, respectively. (**E**) RMSF (root mean square fluctuation) plot per residue, highlighting increased flexibility of the predicted wing of the wHTH DNA-binding motif between residues 92 and 102.

The three-dimensional structure of the *K. oxytoca* TagR homodimer was predicted using AlphaFold 3 by modeling two TagR polypeptide chains to investigate their interaction as a putative transcriptional regulator (21). The model yielded predicted template modeling scores of 0.79 for pTM and ipTM, indicating a reliable global topology and a dimeric interface. To remove incorrect contacts and clashes and refine the predicted AlphaFold model, an equilibrium molecular dynamics (EMD) simulation was subsequently performed. The secondary structure features a three-helix bundle and a two-stranded antiparallel β-sheet, both of which are structural motifs commonly conserved in this family of transcriptional regulators known as wHTH motif (21). The resulting TagR structure, colored by pLDDT scores, is shown in Fig. 1B.

The structural stability of the TagR homodimer was evaluated using a 40 ns EMD simulation (Fig. 1B). The refined final model is presented in Fig. 1C. Protein stability was assessed by analyzing RMSD profiles and inter-chain interaction energy throughout the simulation (Fig. 1B, bottom plot). RMSD values for both chains stabilized during the final 20 ns, with standard deviations of 0.33 Å for chain A and 0.39 Å for chain B, indicating structural stabilization. Additionally, the inter-chain interaction energy remained constant at −678.83 kcal/mol, supporting a robust and persistent interaction between TagR monomers.

RMSF analysis was conducted to characterize residue-level flexibility during the simulation (Fig. 1D). The RMSF profile identified a highly flexible segment spanning residues 92–102 in both chains. This segment corresponds to the predicted DNA-binding motif of TagR. Increased flexibility in this region is consistent with the apo form of the protein, in which the absence of bound DNA permits greater mobility of the DNA-recognition region.

### Conservation of the wHTH DNA-binding motif in TagR protein

**Fig 2:**
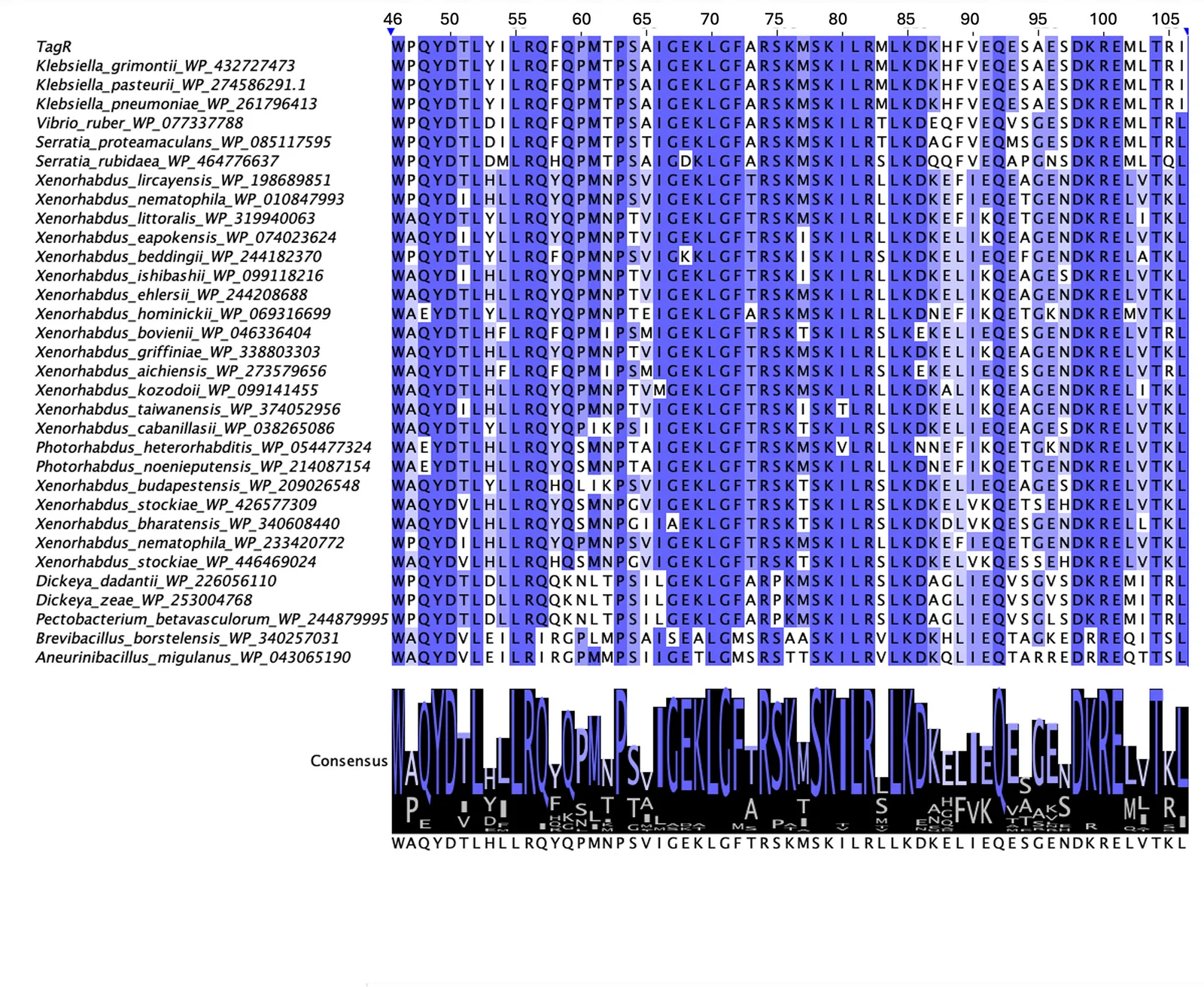
Multiple sequence alignment of the predicted winged helix-turn-helix (wHTH) DNA-binding domain of TagR with representative MarR family transcriptional regulators. The alignment comprises residues 46–106 of TagR and the corresponding regions of homologous MarR family proteins with the highest sequence identity. Amino acid positions are numbered according to the TagR sequence. Residues are colored based on sequence conservation, and the consensus sequence is displayed below the alignment.

The conservation of the wHTH motif in the TagR protein was assessed by performing a multiple sequence alignment of representative MarR family transcriptional regulators from diverse bacterial species, including other *Klebsiella* species (Fig. 2). This alignment demonstrates a high degree of sequence conservation across residues 46–106, as numbered in TagR. Residues critical for the architecture of the wHTH domain, particularly those forming the hydrophobic core (23, 55–57), are strongly conserved in TagR. Indeed, 44.3% of amino acids of such motif are hydrophobics. Furthermore, the motif is highly conserved among *Klebsiella* species, which supports the functional classification of TagR as a member of the MarR family.

### Experimental validation of TagR dimerization and stability

**Fig 3:**
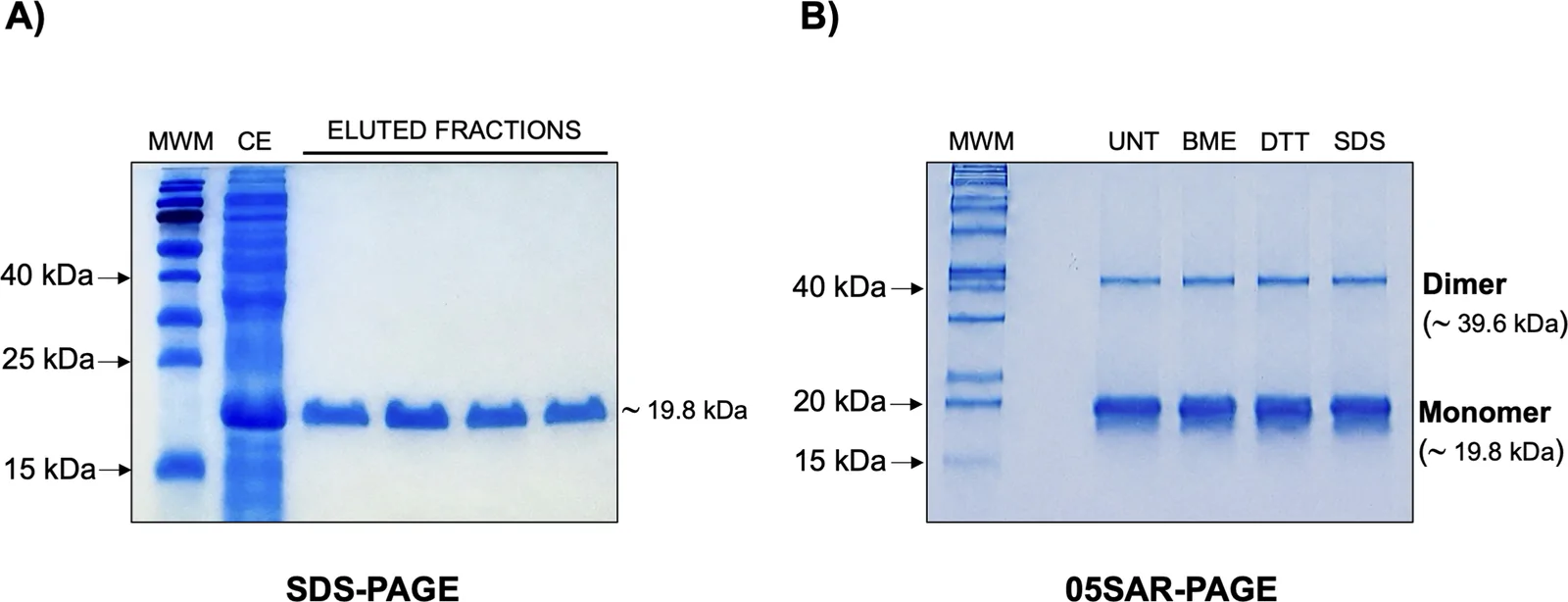
Experimental validation of His_6_-TagR purity and dimer stability. (**A**) SDS-PAGE analysis of recombinant His_6_-TagR purified by Ni-NTA affinity chromatography. Crude extract (CE) and eluted fractions are presented. Eluted fractions display a single predominant protein band at the expected molecular mass of His_6_-TagR (~19.8 kDa). (**B**) 05SAR-PAGE analysis of purified His₆-TagR. Samples were analyzed untreated (UNT) or following treatment with β-mercaptoethanol (BME), dithiothreitol (DTT), or SDS prior to electrophoresis. Monomeric (~19.8 kDa) and dimeric (~39.6 kDa) species are indicated. MWM, molecular weight marker.

His₆-TagR was expressed in *E. coli* and purified by Ni-NTA affinity chromatography. SDS-PAGE analysis showed a single predominant protein band at approximately 19.8 kDa, with no detectable contaminants. This observation aligns with the expected molecular mass of His_6_-TagR and confirms that the preparation is highly purified and appropriate for subsequent biochemical analyses (Fig. 3A).

To assess the oligomeric state predicted by structural modeling, purified His_6_-TagR was analyzed using 05SAR-PAGE. This technique uses 0.05% sarkosyl, a mild anionic detergent that preserves non-covalent protein-protein interactions and facilitates the detection of oligomeric species (41). Under these conditions, His_6_-TagR migrated as two species with molecular masses of approximately 19.8 and 39.6 kDa, corresponding to the expected monomeric and dimeric forms, respectively (Fig. 3B).

To further evaluate dimer stability, purified His₆-TagR was treated with β-mercaptoethanol, DTT, or SDS prior to electrophoresis. The dimeric species remained detectable after treatment with β-mercaptoethanol and DTT, indicating that dimer formation does not depend on disulfide bonds. The persistence of the dimeric band following SDS treatment demonstrates substantial resistance to detergent-induced dissociation (Fig. 3B) and supports the bioinformatic prediction that TagR forms a highly stable dimeric assembly, consistent with a robust dimeric interface.

### Genomic organization of the *til* pathogenicity island, the NRPS operon, and the adjacent regulator TagR

**Fig 4:**
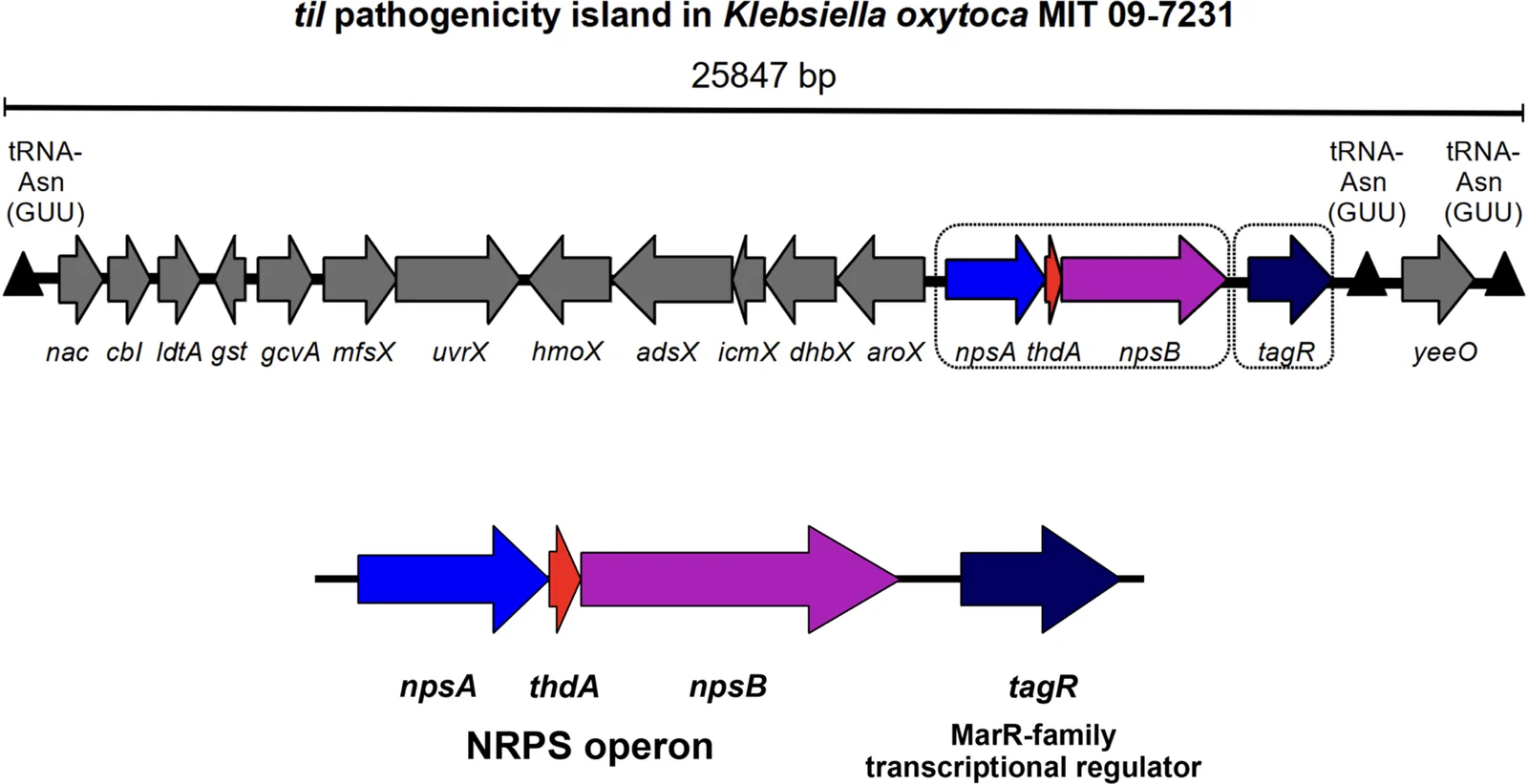
Genomic organization of the *til* pathogenicity island and the NRPS operon in *K. oxytoc*a MIT 09-7231. The upper panel illustrates the genetic organization of the *til* pathogenicity island, emphasizing the position of the *tagR* gene adjacent to the NRPS operon. The lower panel depicts a schematic of the NRPS operon, which includes *npsA*, *thdA*, and *npsB*, as well as the neighboring *tagR* gene that encodes a predicted MarR family transcriptional regulator. Arrow direction indicates gene orientation.

The *til* pathogenicity island of *K. oxytoca* MIT 09-7231 spans 25,847 base pairs, contains 17 predicted genes, and is flanked by asparagine tRNA genes (Fig. 4). Within this island, the nonribosomal peptide synthetase (NRPS) operon consists of the genes *npsA*, *thdA*, and *npsB*, which are oriented in the same direction and form a single transcriptional unit. Adjacent to the operon, the *tagR* gene encodes a predicted MarR family transcriptional regulator. The close genomic proximity of *tagR* to the NRPS biosynthetic genes indicates a possible regulatory relationship, highlighting the need for further investigation into its role in operon expression.

### TagR functions as a repressor of the NRPS operon

**Fig 5:**
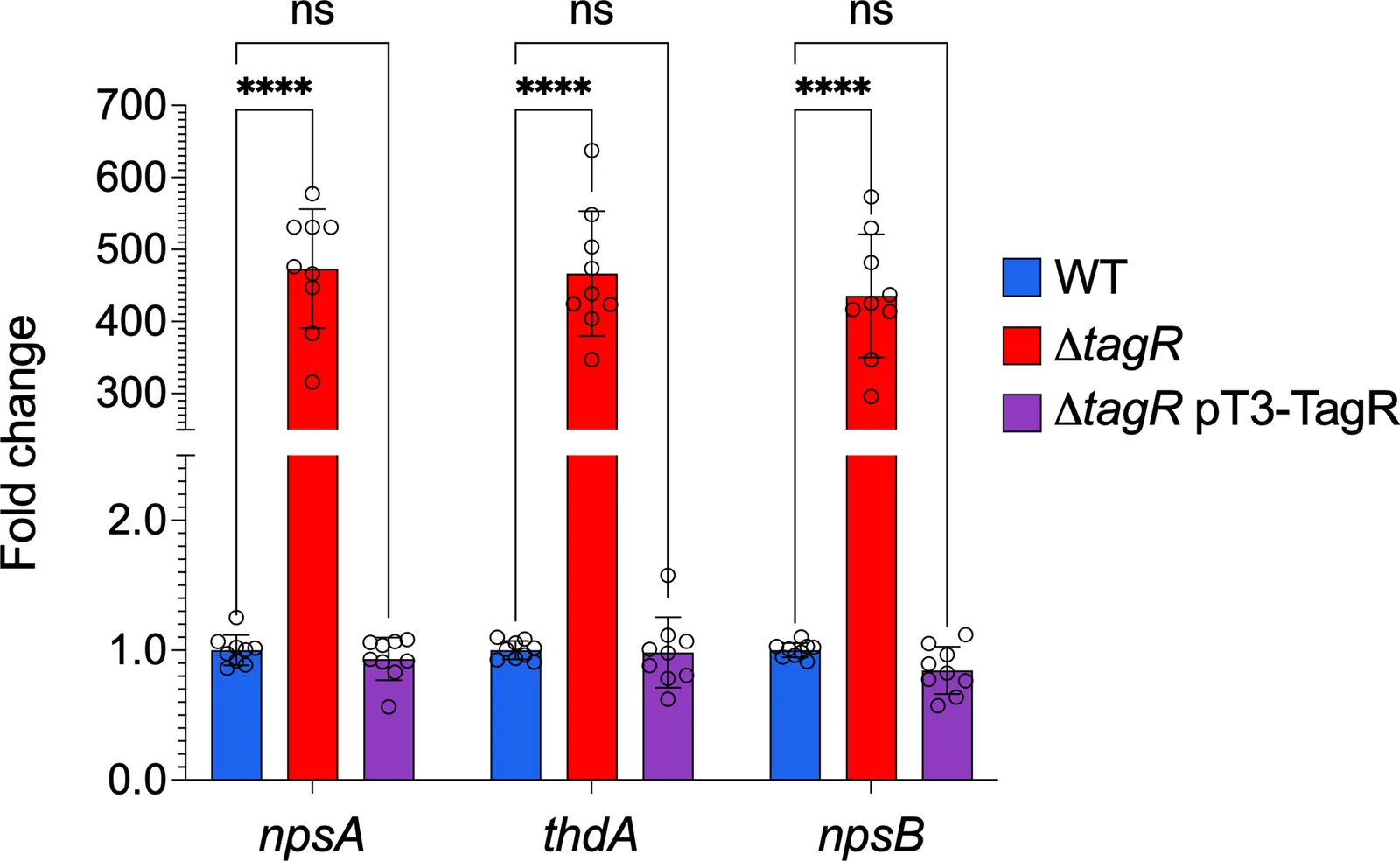
TagR acts as a negative regulator of NRPS operon transcription. Relative expression levels of *npsA*, *thdA*, and *npsB* were quantified by RT-qPCR in wild-type (WT), Δ*tagR* mutant, and complemented (Δ*tagR* pT3-TagR) strains after cultivation in TLB medium. Transcript abundance was normalized to the WT strain. Bars indicate mean values ± standard deviation from three independent biological replicates performed in triplicate. Statistical significance was assessed using one-way ANOVA, followed by Tukey’s comparison test (****P < 0.0001; ns, not significant).

Based on the genomic organization of *tagR* adjacent to the NRPS operon, we next investigated whether TagR regulates operon transcription by determining the transcriptional levels of TM/TV biosynthetic genes using RT-qPCR in the toxigenic *K. oxytoca* strain MIT 09-7231. Deletion of *tagR* resulted in a substantial increase in gene expression, with *npsA*, *thdA*, and *npsB* exhibiting approximately 473-fold, 467-fold, and 436-fold higher transcript levels, respectively, compared to the wild-type strain (Fig. 5). Complementation of the mutant strain with the low-copy-number plasmid pT3-TagR restored transcriptional levels to those observed in the wild-type background. These findings support that TagR acts as a negative regulator by repressing genes involved in TM/TV biosynthesis within the NRPS operon.

Importantly, no apparent growth defects were observed in either the Δ*tagR* or Δ*npsA* mutant relative to the wild-type strain under the experimental conditions used in this study. Therefore, the differences in gene expression observed among the strains are unlikely to result from alterations in bacterial growth.

### Direct interaction between TagR and the regulatory region of the NRPS operon

**Fig 6:**
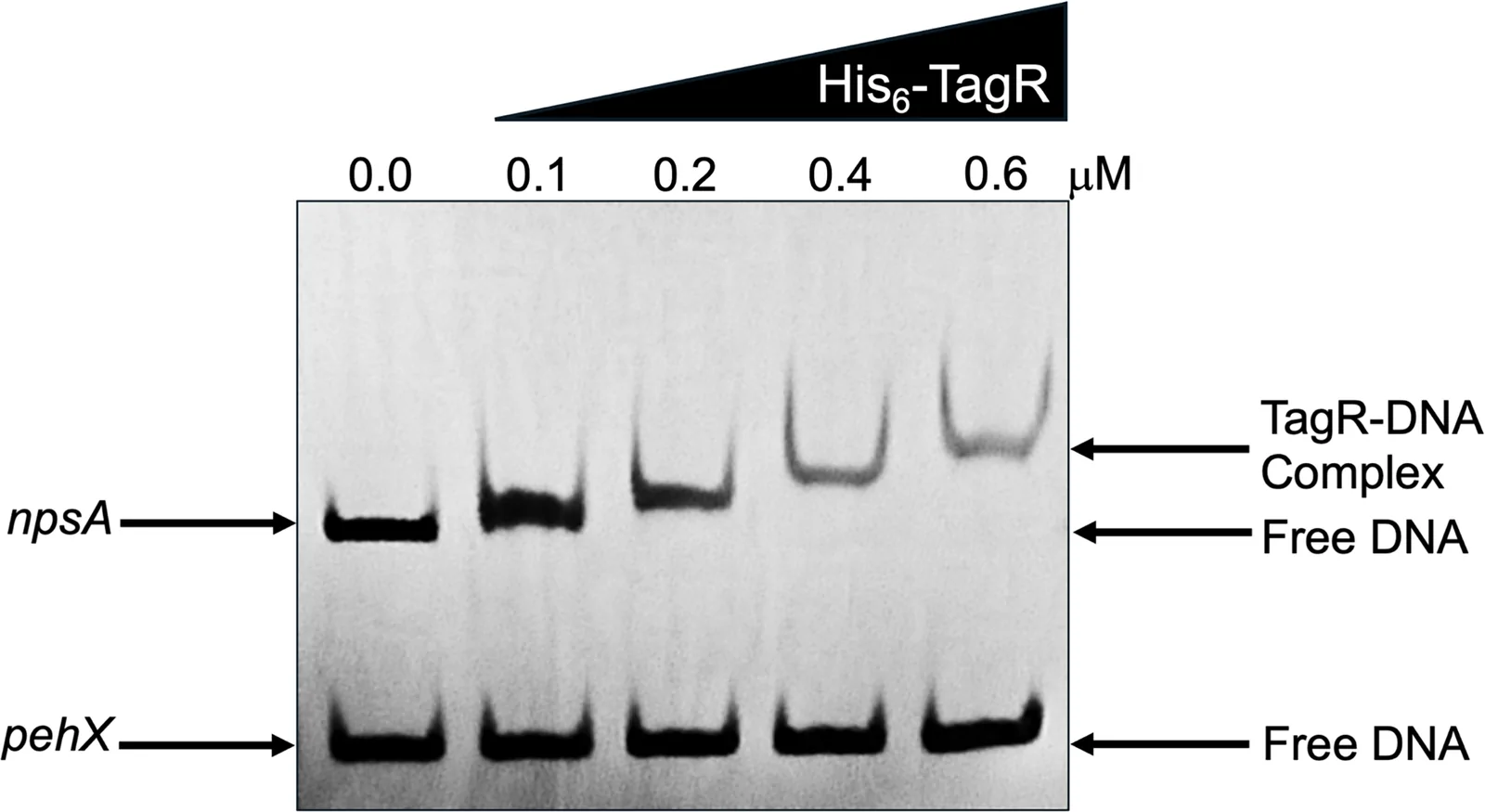
Direct interaction of TagR with the regulatory region of the NRPS operon. Electrophoretic mobility shift assays (EMSAs) were conducted to assess the DNA-binding activity of purified recombinant His_6_-TagR with the upstream regulatory region of the NRPS operon. A DNA fragment from the *K. oxytoca pehX* coding region served as a negative control to evaluate binding specificity. Each reaction contained 100 fmol of DNA probe incubated with increasing concentrations of purified His_6_-TagR protein. Protein–DNA complex formation led to reduced electrophoretic mobility of the NRPS regulatory fragment, while no mobility shift was detected with the negative-control fragment. DNA bands were visualized using ethidium bromide staining. Arrows denote free DNA and TagR–DNA complexes.

EMSAs were conducted using purified His_6_-TagR recombinant protein and DNA fragments corresponding to the regulatory region upstream of the *npsA* gene, the first gene of the NRPS operon, to assess direct interaction. Incubation with 0.2, 0.4, and 0.6 μM His_6_-TagR resulted in a clear mobility shift of the NRPS regulatory fragment, indicating the formation of a protein–DNA complex (Fig. 6).

Binding specificity was evaluated by including a DNA fragment corresponding to the *pehX* gene coding region as a negative control in parallel reactions. No mobility retardation was observed with this fragment, indicating that TagR specifically recognizes sequences within the NRPS regulatory region, rather than binding nonspecifically to DNA. Together, these findings provide strong evidence that TagR functions as a direct transcriptional repressor of the NRPS operon by physically interacting with its upstream regulatory region.

### Identification of the TagR DNA-binding site within the *npsA* regulatory region

**Fig 7:**
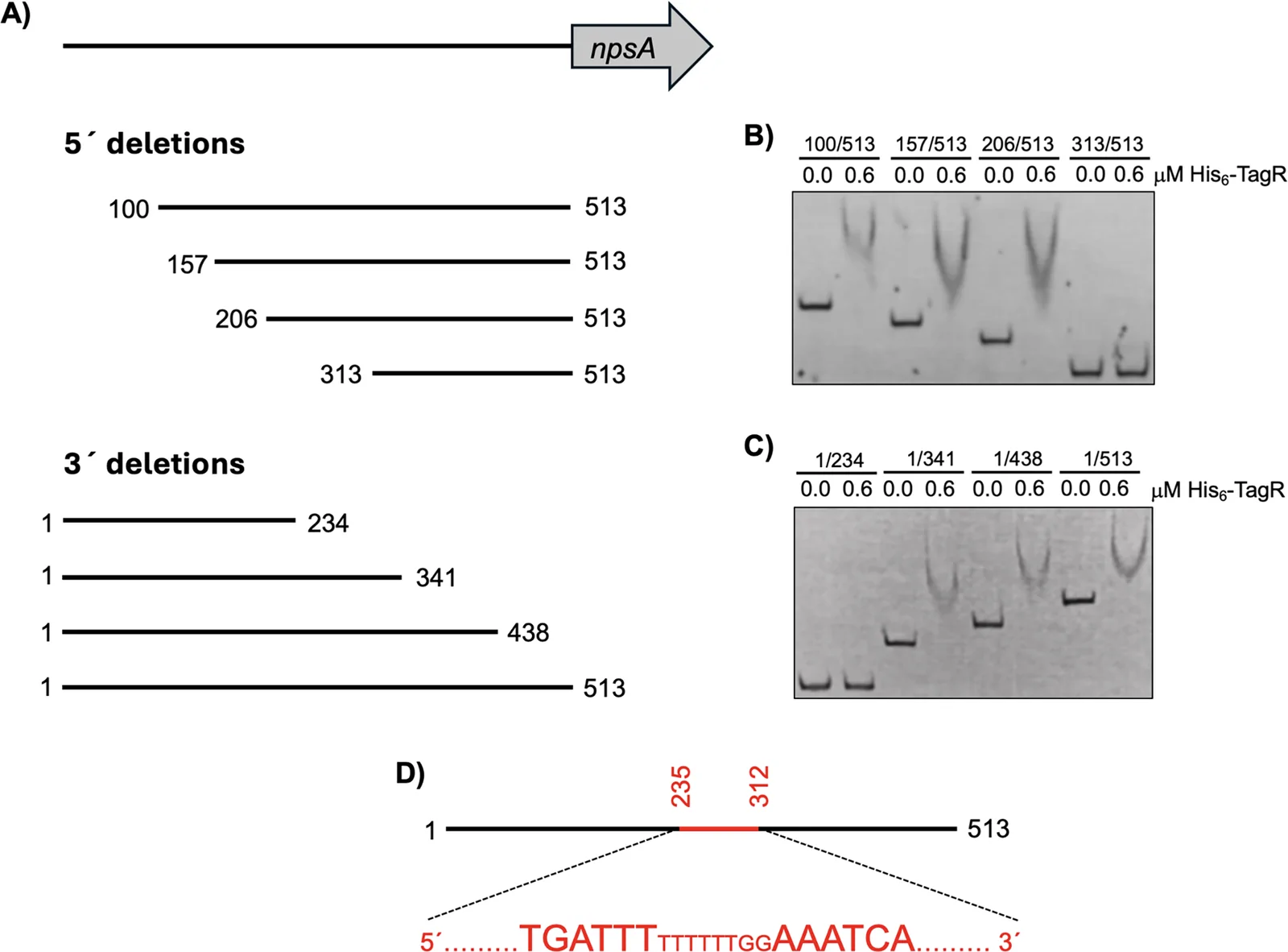
Identification of the TagR-binding region within the *npsA* upstream regulatory sequence using deletion analysis. (**A**) Schematic overview of the 5′ and 3′ deletion fragments derived from the *npsA* upstream regulatory region, which served as probes in electrophoretic mobility shift assays (EMSAs). Nucleotide positions are indicated relative to the 513-bp regulatory fragment. (**B**) EMSA results for 5′ deletion fragments incubated with purified His_6_-TagR (0.6 μM). DNA-protein complexes formed with fragments spanning nucleotides 100–513, 157–513, and 206–513, while no mobility shift was detected with fragment 313–513. (**C**) EMSA results for 3′ deletion fragments incubated with purified His_6_-TagR (0.6 μM). DNA-protein complexes were observed with fragments extending to nucleotides 341, 438, and 513, but not with fragment 1–234. (**D**) Schematic depiction of the minimal DNA region necessary for TagR binding, as determined by combined 5′ and 3′ deletion analyses. The experimentally defined 78-bp region (nucleotides 235–312) contains a predicted inverted-repeat motif consisting of two 6-bp inverted repeats separated by an 8-bp spacer.

A series of 5′ and 3′ deletion fragments from the *npsA* regulatory region were generated and analyzed using EMSA to delineate the DNA region required for TagR binding (Fig. 7A). In the 5′ deletion analysis, His_6_-TagR bound to DNA fragments spanning positions 100–513, 157–513, and 206–513. No detectable mobility shift was observed with the 313–513 fragment (Fig. 7B). These results indicate that sequences upstream of nucleotide 313 are essential for TagR binding.

A complementary 3′ deletion analysis further refined the binding region. TagR did not bind the 1–234 fragment, whereas clear DNA-protein complexes were observed with fragments extending to positions 341, 438, and 513 (Fig. 7C). These findings indicate that the region required for TagR recognition is located downstream of nucleotide 234.

A combined analysis of the 5′ and 3′ deletion experiments localized the TagR-binding region to a 78-nucleotide segment spanning nucleotides 235–312 of the regulatory fragment (Fig. 7D). Analysis of this sequence revealed a putative recognition motif composed of two six-nucleotide inverted repeats separated by an eight-nucleotide spacer: 5′-TGATTT-N_8_-AAATCA-3′.

The results identify a DNA segment necessary for TagR binding and indicate the presence of a putative inverted-repeat motif within this region. The arrangement of this motif aligns with DNA recognition by dimeric MarR family transcriptional regulators, which typically bind palindromic DNA sequences spanning 16–20 nucleotides.

### The TagR-binding site overlaps the predicted *npsA* promoter region

**Fig 8:**
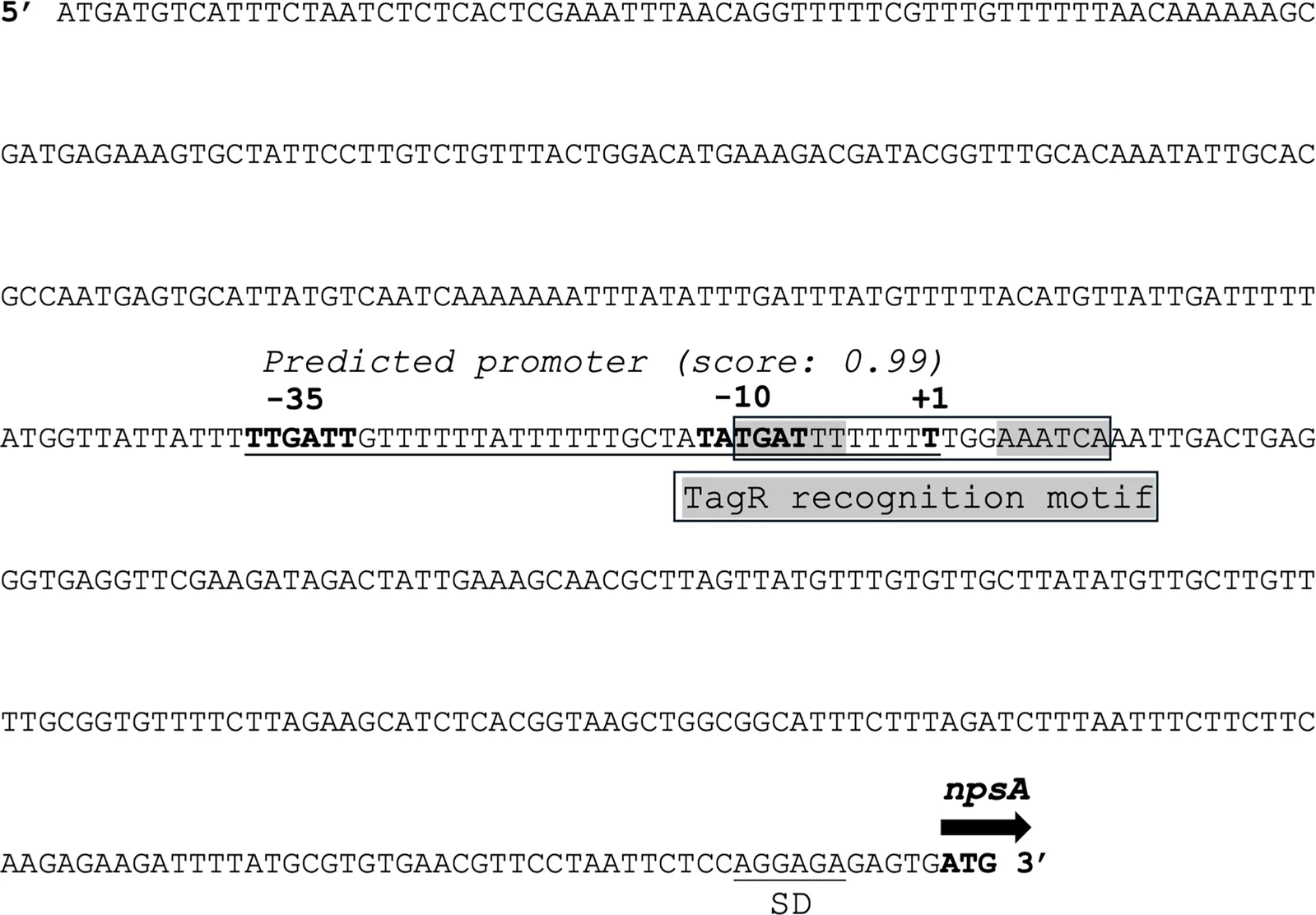
Predicted promoter architecture of the *npsA* regulatory region. The σ^70^ promoter is predicted to contain the −35 and −10 elements, the transcription start site (+1), the Shine-Dalgarno (SD) sequence, and the *npsA* start codon. The 78-bp DNA segment necessary for TagR binding, as identified by deletion analysis, is highlighted in gray. The inverted-repeat motif within this region is also indicated.

To further elucidate the mechanism of TagR-mediated transcriptional repression, the regulatory region upstream of *npsA* was examined for promoter-associated elements. *In silico* promoter prediction identified a putative σ^70^-dependent promoter with a high-confidence score of 0.99, featuring distinct −35 (TTGATT) and −10 (TATGAT) elements and a predicted transcription start site (+1). Additionally, the *npsA* start codon was located downstream of the Shine-Dalgarno sequence (Fig. 8).

Comparison of the experimentally defined TagR-binding site with the predicted promoter architecture revealed substantial overlap between these regions. The first inverted repeat of the TagR recognition motif coincided with the predicted −10 element, while the second inverted repeat extended beyond the transcription start site (+1) into the transcribed region (Fig. 8). Therefore, the TagR-binding sequence encompasses DNA elements essential for transcription initiation.

The location of the recognition motif suggests that TagR binding impedes RNA polymerase access to the promoter or disrupts the formation of the transcription initiation complex. This regulatory arrangement is consistent with mechanisms described for other MarR family members, which frequently repress transcription by binding to operator sequences that overlap core promoter elements (21, 23, 24, 55, 58, 59).

Taken together with the EMSA, deletion mapping, and gene expression analyses, these findings support a model wherein TagR acts as a direct transcriptional repressor of the NRPS operon by occupying a promoter-overlapping operator site and inhibiting efficient transcription initiation.

### Loss of TagR increases cytotoxicity in epithelial cells

**Fig 9:**
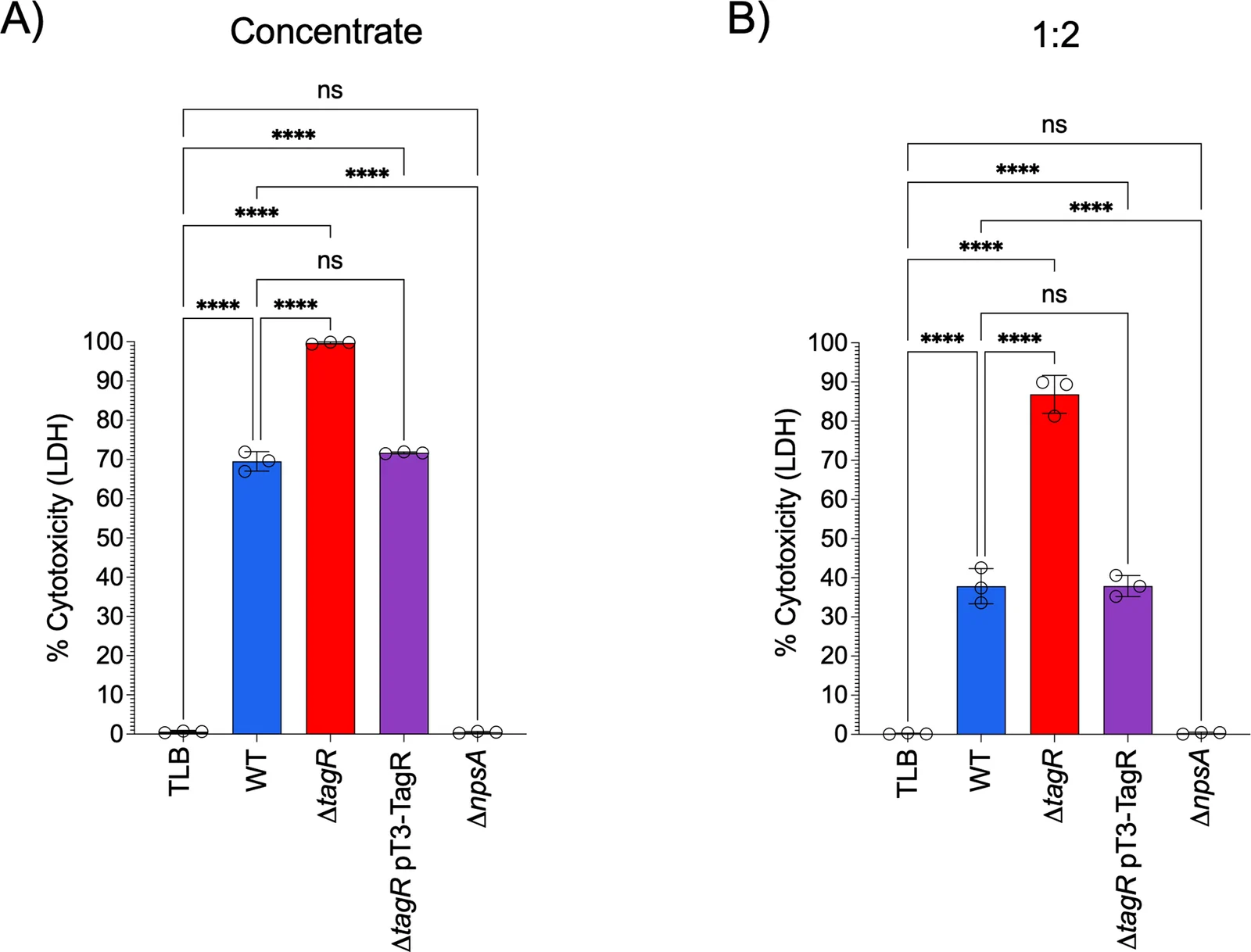
Increased cytotoxicity following TagR deletion in toxigenic *K. oxytoca*. Cytotoxic activity was measured by lactate dehydrogenase (LDH) release assays after exposing HeLa cells to sterile TLB medium (negative control) or to cell-free culture supernatants from the following strains: wild-type (WT), Δ*tagR* mutant, complemented Δ*tagR* pT3-TagR, and Δ*npsA* mutant. All strains were cultured in TLB medium to an OD_600_ of 1.6. (**A**) Cytotoxicity induced by concentrated supernatants. Under these conditions, supernatants from the Δ*tagR* mutant produced cytotoxicity values near the assay’s upper detection limit. (**B**) Cytotoxicity induced by 1:2 diluted supernatants to maintain measurements within the linear detection range. Cytotoxicity percentages were calculated relative to spontaneous and maximum LDH release controls. Data are shown as mean ± standard deviation from three independent biological experiments. Statistical significance was determined using one-way ANOVA, followed by Tukey’s comparison test (****P < 0.0001; ns, not significant).

LDH cytotoxicity assays showed that culture supernatants from the Δ*tagR* mutant exhibited greater cytotoxicity (99.7%) than those from the wild-type strain (69.5%), providing direct evidence that TagR restricts TM/TV-associated virulence under these conditions. Importantly, sterile TLB medium processed identically to the bacterial cultures and included as a negative control did not induce detectable cytotoxicity in HeLa cells after 48 h of incubation, indicating that the culture medium itself did not contribute to epithelial cell damage. In preliminary experiments with undiluted, concentrated supernatants, Δ*tagR*-induced cytotoxicity approached the upper detection limit, further emphasizing the substantial effect of TagR on cytotoxicity regulation. This pronounced response prevented accurate quantitative comparisons between strains (Fig. 9A), underscoring the critical role of TagR in modulating virulence factor activity.

To obtain measurements within the linear detection range, culture supernatants were diluted 1:2, and the experiment was repeated. Following dilution, supernatants from the Δ*tagR* mutant continued to display significantly higher cytotoxicity (86.9%) than those from the wild-type (37.8%) and complemented (37.9%) strains (Fig. 9B). These findings indicate that deletion of *tagR* increases the production of cytotoxic factors associated with the NRPS operon. Complementation of the mutant restored cytotoxicity to wild-type levels, providing further evidence for the regulatory role of TagR in repressing toxin-associated gene expression.

To further confirm the association between the observed cytotoxic phenotype and TM and TV biosynthesis, the Δ*npsA* mutant strain was used as a negative control. Since *npsA* encodes an essential component of the NRPS biosynthetic machinery, disruption of this gene abolishes the production of both enterotoxins (9, 11). Supernatants from the Δ*npsA* mutant did not induce detectable HeLa cell death under the experimental conditions and exhibited cytotoxicity levels comparable to those observed with the TLB control. In contrast to the cytotoxic effects observed with the wild-type and Δ*tagR* strains, the lack of cytotoxicity in the Δ*npsA* mutant provides strong evidence that epithelial cell damage in toxigenic *K. oxytoca* depends directly on the production of TM and TV. These results further support the role of the NRPS operon in the virulence-associated cytotoxic phenotype of this pathogen.

The Δ*tagR* and Δ*npsA* mutants exhibited growth rates comparable to the wild-type strain under the specified experimental conditions. Consequently, the observed differences in cytotoxicity among the strains are likely due to the genetic modifications rather than variations in bacterial growth.

## DISCUSSION

This study identifies TagR as a novel MarR family transcriptional regulator that directly represses the NRPS operon responsible for the biosynthesis of TM and TV in toxigenic *K. oxytoca*. Elucidation of the transcriptional mechanisms governing enterotoxin production establishes TagR as a central negative regulator of virulence-associated gene expression.

MarR family regulators are widely distributed among both gram-negative and gram-positive bacteria, where they participate in adaptive responses to environmental stress, antimicrobial resistance, metabolism, and virulence regulation (23, 60). Members of this family typically function as homodimers that recognize promoter or intergenic DNA regions through a conserved wHTH DNA-binding motif and most commonly act as transcriptional repressors (21). Structural prediction and equilibrium molecular dynamics analyses strongly support the classification of TagR as a member of the MarR family. The predicted protein displays the characteristic α-helical and β-sheet architecture conserved among MarR family regulators, whereas the stable inter-chain interaction energy and low RMSD fluctuations observed during molecular dynamics simulations indicate that the TagR homodimer adopts a structurally stable conformation compatible with DNA binding (61).

The structural assignment of TagR is further reinforced by the conservation of its wHTH DNA-binding motif. Multiple sequence alignment demonstrated that residues forming the hydrophobic core and the overall architecture of this domain are highly conserved among representative MarR family regulators, particularly within *Klebsiella* species. Conservation of these structurally important residues is a defining feature of functional MarR proteins because the hydrophobic core is essential for maintaining the folding, stability, and DNA-binding competence of the wHTH domain (23, 55–57). The high degree of conservation observed in TagR therefore provides evolutionary support for its structural classification and is consistent with its experimentally demonstrated DNA-binding activity.

Although the overall structure of TagR remained stable throughout the simulations, RMSF analysis revealed increased flexibility within the predicted DNA-binding region. Similar dynamic behavior has been reported for several MarR family regulators in their apo conformations, where localized flexibility facilitates adaptation to operator recognition and ligand-induced conformational changes (62). Such conformational plasticity is considered an intrinsic feature of MarR family proteins because subtle rearrangements within the wHTH domain modulate DNA-binding affinity in response to regulatory signals. Accordingly, TagR may undergo conformational changes upon interaction with the NRPS regulatory region or following exposure to environmental or host-derived signals that influence its repressor activity. Identification of these potential regulatory ligands will be important for understanding how enterotoxin biosynthesis is modulated during bacterial adaptation and infection.

The structural predictions were further corroborated by biochemical evidence demonstrating that TagR forms a stable homodimer *in vitro*. Analysis by 05SAR-PAGE revealed the simultaneous presence of monomeric and dimeric species, whereas treatment with β-mercaptoethanol, DTT, or SDS failed to disrupt the dimeric complex, indicating that dimer formation is independent of disulfide bonds and highly resistant to detergent-induced dissociation. These observations are consistent with the well-established behavior of MarR family regulators, which generally function as homodimeric DNA-binding proteins whose oligomeric state is essential for the recognition of palindromic operator sequences (21, 23, 55). Together, the structural predictions, evolutionary conservation of the wHTH motif, molecular dynamics analyses, and biochemical demonstration of homodimer formation support the classification of TagR as a canonical MarR family transcriptional regulator.

A major finding of this study is the substantial increase in NRPS operon transcription following *tagR* deletion. The Δ*tagR* mutant exhibited several hundred-fold increases in the expression of *npsA*, *thdA*, and *npsB*, demonstrating that TagR exerts exceptionally strong negative control over the enterotoxin biosynthetic machinery. Restoration of transcriptional repression in the complemented strain further confirms the specificity of this regulatory effect. Collectively, these results establish TagR as a potent transcriptional repressor that maintains strict control over TM and TV biosynthesis under non-inducing conditions.

The identification of TagR expands the regulatory network controlling NRPS operon expression in toxigenic *K. oxytoca*. Previous studies have demonstrated that the global regulators CRP, Lrp, Fis, and IHF, together with the quorum-sensing regulator SdiA, positively regulate NRPS operon transcription and promote TM/TV production (3, 4, 6, 7). In contrast, the response regulator OmpR negatively regulates this pathway (5). The present study identifies TagR as an additional negative regulator and the first MarR family transcriptional regulator implicated in the control of enterotoxin biosynthesis in *K. oxytoca*. These results support a regulatory model in which NRPS operon expression is governed by multiple transcriptional regulators that integrate nutritional, physiological, environmental, and interspecies signaling cues. Incorporation of TagR into this framework underscores the complexity of the transcriptional network controlling virulence-associated gene expression in toxigenic *K. oxytoca* and suggests that precise regulation of the NRPS operon is required to balance toxin production with bacterial physiology.

EMSAs provided direct experimental evidence that TagR specifically recognizes the upstream regulatory region of the NRPS operon. The absence of mobility shifts when the *pehX* sequence was used as a negative-control DNA fragment demonstrates that the observed interaction is sequence-specific rather than the result of nonspecific DNA association. Direct recognition of promoter regions by homodimeric MarR family regulators represents the canonical mechanism by which these proteins repress transcription of their target genes (30). Therefore, the EMSA results provide the molecular basis for the transcriptional repression observed in the wild-type strain and establish TagR as a direct regulator of the NRPS operon rather than an indirect component of the regulatory network.

Deletion analysis of the *npsA* upstream regulatory region further refined the molecular mechanism of TagR-mediated repression. Progressive 5′- and 3′-terminal truncations localized the minimal TagR-binding region to a discrete 78-bp fragment containing two inverted repeats separated by an 8-bp spacer. Such inverted-repeat architectures are characteristic binding sites for MarR family regulators, whose homodimeric organization enables each monomer to recognize one half-site of a palindromic operator sequence through symmetrical protein-DNA interactions (21, 23, 24, 55). The localization of the TagR-binding site within this conserved structural arrangement is consistent with the canonical DNA-recognition mechanism described for other members of the MarR family and supports the functional significance of the experimentally identified operator.

The identification of two inverted repeats also provides structural support for the experimentally demonstrated homodimerization of TagR. MarR family regulators generally bind DNA as symmetric dimers, with each DNA-binding domain contacting one half-site of the operator. Consequently, the palindromic organization of the TagR recognition sequence is consistent with the biochemical and structural evidence presented in this study and suggests that both DNA-binding domains contribute cooperatively to operator recognition. Such cooperative interactions are known to increase DNA-binding affinity and transcriptional repression by stabilizing the regulator-DNA complex (23, 55).

Notably, the mapped TagR operator overlaps the predicted −10 promoter element and extends across the transcription start site of the NRPS operon. This genomic arrangement strongly suggests that TagR represses transcription through steric occlusion of RNA polymerase binding and/or inhibition of transcription initiation. Similar promoter architectures have been described for numerous MarR family repressors, whose operator sequences frequently overlap essential promoter elements to physically prevent productive interactions between RNA polymerase and the promoter DNA (23, 55, 58, 59). Although additional approaches, such as DNase I footprinting or high-resolution structural analyses, would precisely define the protein-DNA contacts, the combined promoter analysis, EMSA experiments, and deletion mapping strongly support a model in which TagR directly represses NRPS operon transcription by preventing efficient transcription initiation.

This mechanistic model provides a straightforward explanation for the remarkable transcriptional derepression observed in the Δ*tagR* mutant. Removal of the TagR repressor abolishes occupancy of the operator region, thereby allowing unrestricted access of RNA polymerase to the NRPS promoter and resulting in the several hundred-fold increase in *npsA*, *thdA*, and *npsB* transcription observed in the mutant strain. The restoration of wild-type transcription levels following genetic complementation further demonstrates that the observed phenotype is specifically attributable to the loss of TagR, rather than to secondary effects associated with the mutation. Collectively, these findings establish a direct mechanistic link between operator recognition by TagR and transcriptional control of the NRPS biosynthetic operon.

Beyond defining the regulatory mechanism of TagR, these findings substantially advance current understanding of transcriptional regulation within the *til* pathogenicity island. Previous studies have identified several global regulators that modulate NRPS operon expression in response to nutritional status, quorum sensing, or environmental conditions (3–7). In contrast, the present study identifies the specific cis-regulatory element recognized by a dedicated local repressor and provides direct biochemical evidence linking operator recognition with repression of enterotoxin biosynthesis. Together, these observations suggest that NRPS operon expression is controlled through hierarchical integration of global regulatory pathways with promoter-specific repression mediated by TagR, thereby ensuring precise modulation of enterotoxin production under changing environmental conditions.

An additional major finding of this study is that the loss of TagR-mediated repression directly increases bacterial cytotoxicity. The Δ*tagR* mutant exhibited significantly greater cytotoxic activity than the wild-type strain, whereas complementation restored cytotoxicity to basal levels. Because TM and TV are the principal cytotoxins produced by toxigenic *K. oxytoca*, these findings strongly support a model in which derepression of the NRPS operon leads to enhanced enterotoxin biosynthesis and, consequently, increased epithelial damage.

The marked reduction in cytotoxicity observed in the Δ*npsA* mutant provides additional genetic evidence linking the observed phenotype to the NRPS-dependent enterotoxin biosynthetic pathway. Since *npsA* encodes an essential component of the nonribosomal peptide synthetase complex required for TM and TV production, disruption of this gene effectively abolishes toxin biosynthesis despite the presence of the remaining regulatory network. Although TM and TV were not directly quantified in the present study, the combined evidence obtained from transcriptional analyses, promoter-binding assays, and cytotoxicity experiments strongly supports the conclusion that TagR-mediated repression limits enterotoxin-associated cytotoxicity by controlling NRPS operon expression. Direct quantification of TM and TV by liquid chromatography-mass spectrometry would provide valuable quantitative confirmation of this regulatory relationship and represents an important direction for future investigation.

These findings are particularly relevant in the context of, where excessive production of TM and TV directly causes epithelial injury and intestinal inflammation (2, 9, 10). Tight transcriptional regulation of enterotoxin biosynthesis is therefore likely to be essential not only for successful host colonization but also for balancing the considerable metabolic burden associated with secondary metabolite production. In this regard, TagR may function as part of a broader regulatory network that coordinates toxin biosynthesis with bacterial physiology and environmental adaptation, thereby allowing toxigenic *K. oxytoca* to appropriately adjust virulence gene expression during different stages of host colonization and infection.

An intriguing observation is that *tagR* is located within the *til* pathogenicity island, along with the NRPS biosynthetic genes it regulates. The physical linkage between a dedicated transcriptional regulator and its target biosynthetic operon suggests that these genetic elements may have been acquired and maintained as a functional regulatory module during horizontal gene transfer. Similar co-localization of pathway-specific regulators with horizontally acquired virulence determinants has been described in several bacterial pathogens, in which coordinated regulation contributes to the efficient expression of pathogenic traits following the acquisition of genomic islands (63–66). Although comparative evolutionary analyses will be required to test this hypothesis, the genomic organization of the *til* pathogenicity island is consistent with the idea that TagR co-evolved with the enterotoxin biosynthetic machinery to ensure precise transcriptional control of toxin production.

Despite establishing TagR as a direct transcriptional repressor of the NRPS operon, several important questions remain unresolved. The environmental signals that modulate TagR activity have not yet been identified. Many MarR family regulators respond to oxidative stress, antibiotics, metabolic intermediates, reactive oxygen species, or host-derived compounds through ligand-induced conformational changes that alter their DNA-binding affinity (21–23). Whether TagR senses similar stimuli during intestinal colonization remains unknown and represents an important avenue for future investigation. Identification of these regulatory signals will provide valuable insight into how toxigenic *K. oxytoca* coordinates enterotoxin production in response to changing environmental conditions within the host.

It also remains unclear whether TagR exclusively regulates the NRPS operon or participates in broader transcriptional networks. Numerous MarR family proteins function as pleiotropic or global regulators that coordinate multiple physiological pathways associated with stress adaptation, metabolism, antimicrobial resistance, and virulence (20, 21, 23, 60). Future transcriptomic analyses, chromatin immunoprecipitation coupled with high-throughput sequencing, or genome-wide DNA-binding studies will be valuable for defining the complete TagR regulon and determining whether additional virulence-associated genes are directly controlled by this regulator.

In conclusion, this study identifies TagR as a previously uncharacterized MarR family transcriptional repressor that directly controls expression of the NRPS operon responsible for TM and TV biosynthesis in toxigenic *K. oxytoca*. Integration of structural prediction, molecular dynamics simulations, evolutionary conservation, biochemical characterization, promoter-binding analyses, genetic approaches, and functional cytotoxicity assays demonstrates that TagR represses enterotoxin biosynthesis through direct recognition of the NRPS promoter region. Loss of TagR results in marked derepression of toxin biosynthetic genes and enhanced epithelial cytotoxicity, underscoring its central role in controlling NRPS operon expression. Collectively, these results expand current understanding of the transcriptional mechanisms governing enterotoxin biosynthesis in toxigenic *K. oxytoca*, identify TagR as the first MarR family transcriptional regulator demonstrated to directly repress the NRPS operon, and provide a foundation for future studies aimed at identifying the environmental or physiological signals that modulate TagR activity.
